# Individual and contextual factors predicting self-reported malaria among adults in eastern Indonesia: findings from Indonesian community-based survey

**DOI:** 10.1186/s12936-019-2758-2

**Published:** 2019-04-04

**Authors:** Pandji Wibawa Dhewantara, Mara Ipa, Mutiara Widawati

**Affiliations:** 1 Pangandaran Unit for Health Research and Development, National Institute of Health Research and Development, Ministry of Health of Indonesia, Pangandaran, West Java 46396 Indonesia; 2 School of Veterinary Science, University of Queensland, Gatton, QLD 4343 Australia

**Keywords:** Multilevel analysis, Malaria, Indonesia, Riskesdas, Papua, Maluku

## Abstract

**Background:**

Malaria is still an important parasitic infectious disease that affecting poor and vulnerable communities in many developing countries, including Indonesia. During the period of 2010–2017, there have been approximately 2.2 million confirmed malaria cases reported across Indonesia. This study aimed to identify individual, household and village-level factors associated with self-reported malaria among adults more than 15 years of age in Maluku, West Papua and Papua province.

**Methods:**

This study analysed a subset of the data from nationally representative population-based Indonesian National Basic Health Research (Riset Kesehatan Dasar) (N = 1,027,763 in 294,959 households in 33 provinces) in 2013. Total of 41,079 individuals (20,326 males and 20,753 females) aged ≥ 15 years in 19,269 households in Maluku, West Papua and Papua provinces were included. Participants were interviewed if they ever had been diagnosed and laboratory confirmed of having malaria by physician in the past 12 months. A mixed effects multilevel logistic regression models were developed to assess the associations between socio-demographical variables at individual, household and village level and self-reported malaria.

**Results:**

Individuals aged ≥ 15 years in 701 villages in Maluku (n = 11,919), West Papua (n = 8003) and Papua (n = 21,157) were analysed. In all provinces, gender distribution was equally-represented. The prevalence of self-reported malaria was 4.1% (Maluku), 12.4% (West Papua) and 18.8% (Papua). At the individual level, primary industry workers (OR 1.29, 95% CI 1.15–1.46 [Maluku]; OR 1.17, 95% CI 1.09–1.25 [Papua]) and having higher education were associated with self-reporting malaria (OR 0.67, 95% CI 0.53–0.83 [Maluku]; OR 1.27, 95% CI 1.15–1.40 [Papua]). Household level factors include having bed net and better off wealth index were associated with increased self-reporting malaria among West Papua (OR 1.21; 95% CI 1.09–1.34 and OR 1.38; 95% CI 1.17–1.65, respectively) and Papuan (OR 1.12; 95% CI 1.02–1.23 and OR 1.33; 95% CI 1.11–1.57, respectively) adults. Increased odds of self-reporting malaria was associated with time required to reach healthcare facility (OR 1.30, 95% CI 1.01–1.67 [Maluku]). Contextual village-level characteristics such as living in rural (OR 1.31, 95% CI 1.12–1.54 [Maluku]; OR 1.56, 95% CI 1.17–2.07 [West Papua]), higher community education level (OR 1.28, 95% CI 1.02–1.63 [West Papua]; OR 1.45, 95% CI 1.23–1.72 [Papua]), higher community bed net ownerships (OR 0.59 95% CI 0.45–0.77 [West Papua]) were associated with self-reported malaria.

**Conclusions:**

Factors associated with self-reported malaria were varied between provinces suggesting locally-specific determinants were exist at individual, household and community-level. This study highlights the need for specific interventions by taking into consideration the contextual factors within the region and involving multi-sectoral collaboration between health authorities and related stakeholders (e.g., bureau of education, bureau of public works and infrastructure) to improve designs in planning and intervention strategies to succesfully eliminate malaria in Maluku and Papua.

**Electronic supplementary material:**

The online version of this article (10.1186/s12936-019-2758-2) contains supplementary material, which is available to authorized users.

## Background

Malaria is still an important parasitic infectious disease that affecting poor and vulnerable communities in many developing countries. Globally, there were more than 200 million cases and 445,000 deaths reported in 2016 [1]. Indonesia is one of the endemic countries where more than 25% of its people still residing in active transmission areas [2]. During the period of 2010–2017, there have been approximately 2.2 million confirmed malaria cases reported across Indonesia [3] and most of these malaria cases were reported from the eastern Indonesia including Papua, West Papua and Maluku [4], where a considerable number of *Anophelines* species and both parasite *Plasmodium falciparum* and *Plasmodium vivax* are still found in this region.

Although there has been a substantial decline in the national annual parasite incidence (API) during 2009 to 2017, the API (per 1000 persons) for those three provinces in 2017 remains high (Papua: 59.00, West Papua: 14.97 and Maluku: 2.30) compared to the national API (0.99/1000 persons) [3, 5]. In 2020, Indonesia is expected to achieve the pre-elimination stage for malaria—which indicated by a reduction of active foci and API < 1 per 1000 people and become one of the malaria-free countries in Southeast Asia by 2030. To achieve these goals, knowledge concerning local risk factors of malaria is essential to guide health authorities for implementing effective intervention strategies especially in that high malaria transmission areas in eastern Indonesia [6]. Yet, determinants of malaria transmission especially in this region such as Maluku and Papua islands are still far from clear.

Malaria transmission involves a complex interaction of factors within the ecosystem including *Plasmodium* parasites, *Anophelines* mosquitoes, human hosts and local socio-ecological conditions. A considerable number of studies in many endemic countries have described a number of malaria risk factors. Malaria was found to be associated with gender, age, occupation and behaviours [7–9]. At household-level, number of inhabitant, household economic condition (e.g., housing and income), insecticide-treated nets (ITNs)/long-lasting insecticidal nets (LLINs) ownerships and the availability of healthcare facilities were determined the level of malaria risk [10–12]. Moreover, ecological condition such as altitude has also been demonstrated as important factor associated with risk of malaria [13–16]. However, the effects of these individual-, household- and environmental factors to malaria risk in Maluku and Papua has not been fully quantified. Although there have been numerous studies on identifying risk factors associated with malaria infection in Indonesia [17–19], knowledge regarding individual, household, and village-level factors associated with malaria especially in both Maluku and Papua are still lacking. Understanding the risk factors at each level is important so that effective resource allocation for local elimination malaria strategies can be implemented.

Individual health outcome is influenced by various factors including the environment, communities, and socioeconomic condition of the area where they lived [20, 21]. An appropriate and advance statistical modelling approach is needed as traditional statistics modeling could not able to fully explain the effect of covariates at each level on the individual outcome and it could potentially lead to the ecological fallacy. To overcome this problem, multilevel modelling approach could be used as it allows for the investigation of the effects of contextual variables measured at the different levels both at micro and macro-level on the individual-level outcome [20, 22–24]. The role of environment and socioeconomic factors on malaria has been conducted in many parts in Indonesia [17–19, 25, 26]. However, there have been limited studies using multilevel approach to determine the effect of micro-level scale factors on the individual malaria infection, especially in Maluku and Papua where malaria is still highly prevalent.

A recent Indonesian nationwide population-based survey, Basic Health Research (Riset Kesehatan Dasar, RISKESDAS) included malaria as one of infectious disease variable and collected household level information which enables us to obtain better understanding on household-level factors associated with malaria, especially in the province of Maluku, West Papua and Papua. Utilizing these data, the objective of this study was to determine social and environmental conditions associated with self-reported malaria among adults ≥ 15 years of age in Maluku, West Papua and Papua province. The result of the study will provide evidence base for better strategies and resource allocation especially in these studied areas.

## Methods

### Description of the study areas

**Fig. 1:**
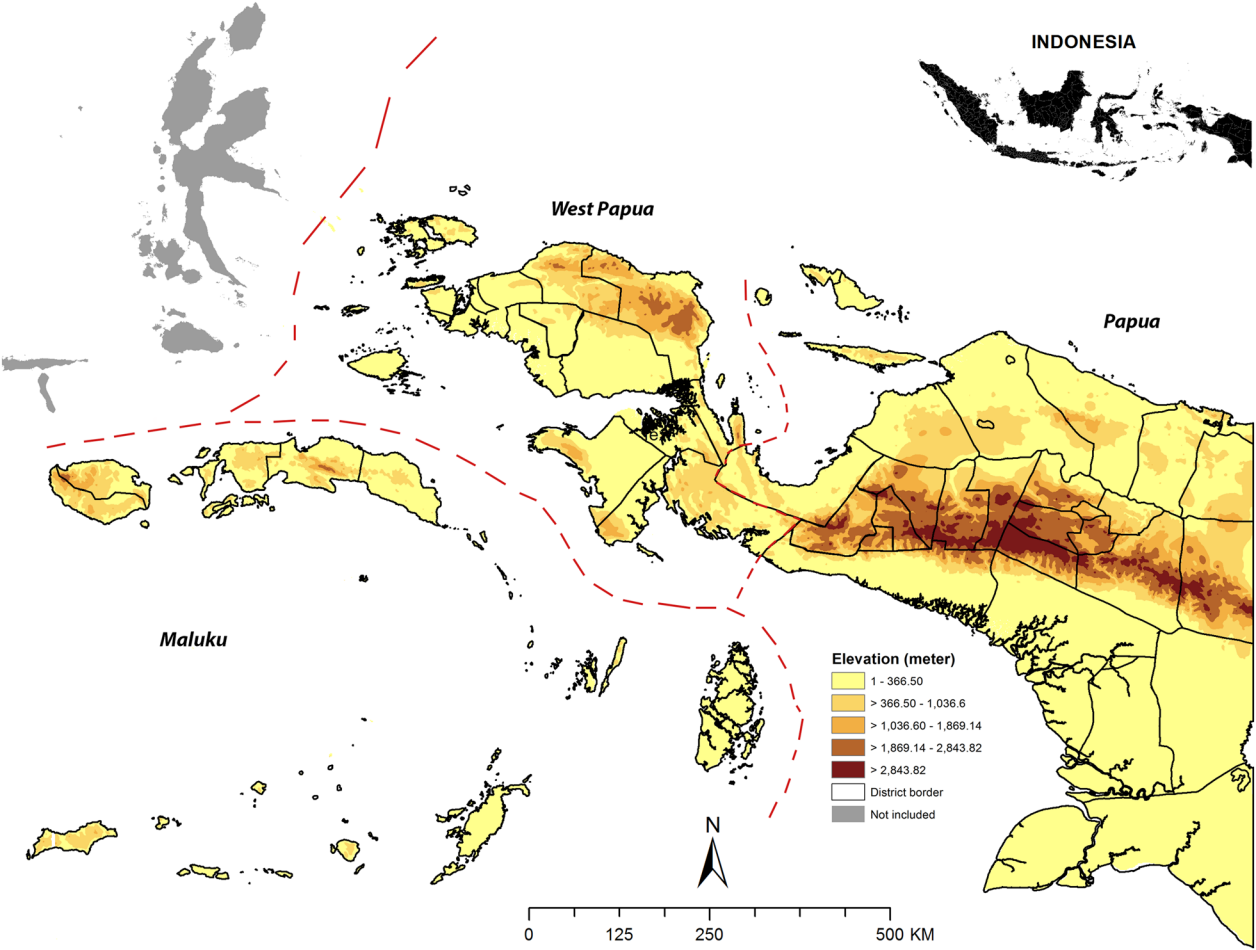
Study sites. Red dash line indicates administrative border. Elevation data were retrieved from digital elevation model (DEM) Global 30 Arc-Second Elevation (GTOPO30) with 1-kilometre spatial resolution from the United States Geological Survey (USGS) Earth Resources Observation and Science (EROS) Center (https://lta.cr.usgs.gov/GTOPO30). The map was created in ArcGIS 10.5.1 software, ESRI Inc., Redlands, CA, USA, (https://www.arcgis.com/features/index.html)

The present study was restricted to three provinces in eastern Indonesia: Maluku, West Papua and Papua province (Fig. 1). Maluku province consisting of 11 districts and it is an archipelago with more than 600 islands stretch between North Maluku and Ceram Sea (north), West Papua province and Arafura Sea (east), East Timor and Timor Sea (east) and the province of Southeast Sulawesi and Banda Sea (west). Maluku province has population of 1.5 million people and area of 54,185 km^2^ with elevation ranging from 3 to 3027 m [27]. Both West Papua and Papua provinces are situated in the west part of the Papua islands which border to Papua New Guinea in the east. West Papua province consisting of 12 districts and 1 municipality, inhabited by more than 800,000 people with area of 99,671 km^2^ [28]. While Papua province consisting 28 district and 1 municipality and it has population of 2.8 million people with an area of 316,553 km^2^ [29]. Papua islands have elevation ranging from 1.17 m to more than 4000 m, with the highest altitude is found in Puncak Jaya district.

### Study design, sampling technique and data collection

This study was a secondary analysis of 2013 Indonesia Basic Health Research (Riset Kesehatan Dasar, RISKESDAS 2013) to investigate the determinants of self-reported malaria in Maluku, West Papua and Papua province. Methodologies used in the RISKESDAS 2013 have been described elsewhere [30]. Briefly, RISKESDAS is a cross-sectional, quinquennial and nationwide community-based survey aimed to evaluate public health indicators for policymaking at national, provincial and district-level. The population of RISKESDAS 2013 was all households in 33 provinces. Household samples were selected through stratified multistage systematic random sampling design and the probability proportional to size (PPS) approach. To illustrate, at the first stage, 30,000 primary sampling units (PSUs) was selected using PPS technique based on the 2010 Indonesia Population Census. Each PSUs contained several census blocks (CBs) which defined as the enumeration areas of the census. The second stage was the selection of CBs at each selected PSU using PPS based on household number estimated from 2010 Indonesia Population Census. The third stage was systematically selected 25 households from selected CBs. Finally, there were 12,000 CBs and 300,000 households selected as sample of RISKESDAS 2013. Exclusion was applied if the CB could not be able to identify or access because of natural disaster, social unrest/conflict, extreme geographic conditions.

Data collection was performed by trained enumerators. These enumerators were visited selected households guided by local health authorities and village leader. Informed consent procedures were followed and information was collected through face-to-face interviews by the enumerators using structured questionnaires. Head of the households and all members were interviewed. Survey participants aged under the age of 15 years were accompanied by a parent or guardian during the interview The questionnaire consisted of several sections including household-level section (e.g., access and health services, pharmacy and traditional health services, mental health, environmental health and housing and economy) and individual-level section (e.g., communicable diseases, non-communicable diseases, injury, oral health, disability, mental health, knowledge-attitude-practices (KAPs), health financing, reproductive health, children’s health and immunization, and physical measurements and biomedical data collections). Of which, malaria was one of the communicable diseases included in the questionnaire. In total, RISKESDAS 2013 had interviewed 294,959 households with 1,027,763 household members in 33 provinces [4].

### Sample

The present study utilized a subset of data of RISKESDAS 2013. The analysis was included sample of respondents aged ≥ 15 years (N = 41,079; 20,326 males and 20,753 females) resided in Maluku (n = 11,919), West Papua (n = 8003) and Papua (n = 21,157). These three provinces were selected since most of the malaria cases were contributed by these three provinces accounting for 80.41% of the total confirmed malaria cases in Indonesia [3, 5]. In addition, these three areas are also representing different level of endemicity in which Maluku represented a medium transmission risk (API 1–5 per 1000 populations while both West Papua and Papua province represented high transmission areas (API > 10 per 1000 and parasite prevalence as high as 50–75%) [5].

### Variables

The outcome variable in this study was self-reported malaria. Each household’s member was asked whether he/she had been diagnosed of having positive laboratory-confirmed malaria by local health providers/physicians in the past 12 months. The response to this question was binary: code 1 (Yes) and 0 (No) was given. In health facilities, malaria was generally confirmed by using rapid diagnostic tests (RDTs) and microscopy. In this survey, the interviewer did not perform any diagnostic tests.

The explanatory variables were defined into three levels: village, household and individual variables. RISKESDAS dataset contains unique data identification (ID) which describe information on household and village where each individual nested, so that the village ID could be identified and extracted. Village-level variables were obtained by aggregating the individual variables into the village-level. The village-level variables included in this study were the proportion of villagers who had access to improved drinking water sources (tap/piped water, boreholes, protected dug wells, protected springs and rainwater collection), bed nets ownership and village-level education. These continuous variables were converted into categorical format: “High” and “Low”. For those three variables, the cut off was defined. Cut-off of 60% for access to improved drinking water was determined based on the national coverage (proportion) of household with improved water sources [4]. While the cut-offs for bed net ownership were varied depending on location. This cut-off was based on the province-specific median proportion of bed-net ownership, which obtained from other analysis of RISKESDAS 2013 [4]. The cut-offs for education were also province-specific, which was based on the average proportion of population aged ≥ 15 years attending high-school for each province which obtained from the Annual Report of Provincial Bureau of Statistics of 2014 (http://www.bps.go.id). Code 1 indicates “High” was given to the village when the proportion of access to improved drinking water, bed net ownership and population attended high-school more than the predefined cut-offs for each variable. In addition, type of residence (urban/rural) and zone (highland, midland and lowland) were included as village-level variable. As elevation data were not available in the RISKESDAS dataset, satellite data digital elevation model (DEM) Global 30 Arc-Second Elevation (GTOPO30) with 1-km spatial resolution obtained from the United States Geological Survey (USGS) Earth Resources Observation and Science (EROS) Center (https://lta.cr.usgs.gov/GTOPO30) was utilized. Administrative boundary maps were obtained from the Bureau of Statistics of Indonesia (*Badan Pusat Statistik*—*Sistem Informasi Layanan Statistik*) (http://www.silastik.bps.go.id). The mean areal elevation values were sampled by ArcGIS 10.5.1 (ESRI Inc., Redlands, CA, USA) using zonal statistics toolkit. Each area was then classified into one of the three zones based on their elevation: lowland (< 200 m), midland (200–1200 m) and highland (> 1200 m).

The household-level variables such as bed net ownership, household size, households’ perception on time needed to travel to the nearest health facilities (categorized into not available, less or more than 30 min) and wealth index (poorest, poorer, middle, richer, richest) were included. In terms of bed net ownership and perception regarding access to the health facilities, the head of the household was asked whether the household owned bed net and how they perceived access to health facilities (in terms of time required to travel to the nearest health services). The wealth index was estimated using principle component analysis (PCA) as described elsewhere [30]. Total 12 variables were used for PCA including housing and sanitation (e.g., source of drinking water, toilet facilities, type of toilet, behaviour of stool disposal), source of energy (e.g., type of light, cooking fuel) and household assets (e.g., motor cycle, television, water heater, 12-kgs gas tube, refrigerator and car ownership). Total 54 models resulted from the PCA. Of which, the best fit model with the highest proportion of variation explained (53.6%) containing 12 variables above was selected [4].

The individual-level variables such as gender, occupation (categorized into three groups: not working, primary [farmers, fishermen] and tertiary sector [civil servants, private workers, army, entrepreneurs] workers and other), level of education attained (none, primary, higher), and variable indicating whether participants slept under insecticide-treated bed net (ITN) last night (dichotomized into yes and no) before the survey conducted were included.

### Statistical analysis

A descriptive analysis was conducted to describe baseline characteristics and weighted proportion of the explanatory variables. Collinearity between covariates was checked using Spearman correlation analyses. Covariates that had strong correlation (Spearman’s rho ≥ 0.9) were not included in the model. Based on the results of correlation analysis, there is no strong correlation detected among covariates. All associations between covariates and self-reported malaria were first examined by bivariate logistic regression. All significant variables with *P* values of Wald test ≤ 0.25 were considered statistically significant [31] and further included in the final multivariate multilevel mixed-effects logistic regression. Multivariate multilevel mixed-effects logistic regression models with individuals nested in households, households nested in villages constructing a three-level model with random intercepts were built. Multilevel analysis is a statistical tool applied to data with nested sources of variability, which involve units at lower level nested within units at a higher level [32, 33]. A three-level model was applied as follows Model 0 (null model) which only contained a random intercept and overall variation in malaria at the village-level. Model 1 contained individual-level covariates, Model 2 contained individual and household-level covariates and full model (Model 3) included individual, household and village-level covariates. In this present paper, only the final model is presented. Results were presented in terms of odds ratio (OR) and 95% confidence intervals (CIs).

Significance was set at *P* < 0.05. Variation of the outcomes at the village-level was described by village-level (random effect) variance and standard error. The percentage of proportional change in variance (PCV) was calculated between the null model and each subsequent model to examine the extent to which the covariates explained the variation in malaria across villages [32]. All statistical analyses were performed in STATA 15.1 (Stata Corp., College Station, TX). To allow for the cluster sampling design of the survey, the ‘*svy*’ command was used. Mixed effect multilevel analysis was performed using ‘*melogit*’ command.

## Results

### Sample characteristics

Table 1 shows the characteristics of the overall study participants included in the analysis. In total, 41,079 individuals aged ≥ 15 years in 701 villages in three provinces in the eastern Indonesia, including Maluku (n = 11,919; 162 villages), West Papua (n = 8003; 135 villages) and Papua (n = 21,157; 404 villages) were included in the analysis. Of all participants in all provinces, gender distribution was equally-represented and the majority of respondents (47.1 to 63.8%) had attained secondary education level. A high proportion of Papuan respondents were involved in primary sector as farmers or fishermen (n = 9358; 42.6%), although half of the respondent in Maluku (n = 5709; 50.5%) and West Papua (n = 3705; 46.5%) were unemployed. Small proportion of participants in Maluku (20.9%) and Papua (27.4%) having slept under a net the previous night before the survey. Bed net ownership at the time of the surveys ranged from 29.2 to 36.2%, with the highest proportion found in West Papua. Household density was relatively high in all provinces. Up to 60–80% of respondents were perceived of having health facilities nearby within 30 min travel. Unlike in Maluku and West Papua, more than half of the respondents in Papua (n = 12,902; 58.5%) had the lowest wealth index. The majority of respondents resided in rural areas (59–71%) and in a village where the proportion of bed net usage among villagers was low (52–63.7%). Majority of respondents were living in a village with good access to drinking water sources (50.1–81.1%), better community education level (57.1–60.1%). Most of respondents in Maluku and Papua were lived in lower highland zone areas (52.1% and 40.4%, respectively) while up 59% of participants were resided in West Papua’s lowland.

**Table 1:** Characteristics of the study participants, adults aged ≥ 15 years, in the three selected provinces in eastern Indonesia

Variable	Maluku N = 11,919 (%^a^)	West Papua N = 8003 (%^a^)	Papua N = 21,157 (%^a^)
Malaria	563 (4.1)	905 (12.4)	3964 (18.8)
Type of malaria			
*P. falciparum*	90 (12.0)	233 (22.3)	1538 (37.5)
*P. vivax*	105 (17.9)	456 (56.9)	1537 (41.0)
*P. falciparum *+* P. vivax*	11 (3.0)	34 (3.5)	183 (5.7)
Other	59 (12.6)	26 (2.1)	193 (2.5)
Not known	298 (54.4)	156 (13.3)	1097 (13.3)
Individual-level			
Gender			
Male	5541 (50.0)	3853 (53.2)	10,932 (52.9)
Female	6378 (50.0)	4150 (46.8)	10,225 (47.1)
Occupation			
Not working	5709 (50.5)	3705 (46.5)	7067 (33.7)
Primary industry workers (farmer, fisherman)	3383 (23.9)	1980 (20.0)	9358 (42.6)
Tertiary industry workers (services-related occupation)	2273 (20.8)	2033 (30.0)	3902 (19.6)
Other (undefined)	554 (4.8)	285 (3.4)	830 (4.1)
Education			
No	532 (3.3)	579 (4.5)	4481 (20.8)
Primary	4501 (33.0)	3224 (32.2)	7438 (32.0)
Secondary	6886 (63.8)	4200 (63.2)	9238 (47.1)
Used ITN last night			
No	8840 (79.1)	3730 (55.3)	13,858 (72.6)
Yes	3079 (20.9)	4273 (44.7)	7299 (27.4)
Household-level			
Bed net ownership			
No	8563 (68.9)	3710 (63.8)	13,949 (70.8)
Yes	3356 (31.1)	4293 (36.2)	7208 (29.2)
Household-density			
Less than 8/m^2^	3435 (31.2)	1713 (22.8)	8449 (45.1)
More than 8/m^2^	8484 (68.8)	6290 (77.2)	12,708 (54.9)
Access to the nearest public health services			
Not available	1035 (8.1)	670 (7.8)	1753 (8.9)
Less than 30 min	9377 (80.0)	6680 (85.1)	12,549 (59.2)
More than 30 min	1507 (11.9)	653 (7.1)	6855 (32.0)
Household wealth index			
Poorest	2129 (14.3)	2893 (25.9)	12,902 (58.5)
Poorer	2253 (17.0)	2118 (23.3)	2717 (12.1)
Middle	2334 (18.6)	1169 (17.5)	1864 (8.8)
Richer	2508 (22.5)	1143 (21.1)	1894 (9.3)
Richest	2695 (27.5)	680 (12.2)	1780 (11.2)
Village-level			
Place of residence			
Rural	7133 (58.7)	5950 (60.9)	15,722 (71.2)
Urban	4786 (41.3)	2053 (39.1)	5435 (28.8)
Proportion of villagers used bed net^b^			
Low	6667 (55.9)	3505 (63.7)	11,011 (52.0)
High	5252 (44.1)	4498 (36.3)	10,146 (48.0)
Proportion of villagers have access to improved water sources^c^			
Low	5305 (44.5)	1722 (18.9)	10,552 (49.9)
High	6614 (55.5)	6281 (81.1)	10,605 (50.1)
Proportion of villagers had secondary education level^d^			
Low	4756 (39.9)	3170 (42.9)	8608 (40.7)
High	7163 (60.1)	4833 (57.1)	12,549 (59.3)
Zone			
Lowland (< 200 m)	5816 (47.9)	4704 (58.8)	5200 (24.6)
Midland (200–1200 m)	6103 (52.1)	2151 (26.8)	8546 (40.4)
Upper highland (> 1200 m)	0 (0.0)	1148 (14.4)	7411 (35.0)

*ITN* insecticide-treated net

^a^Weighted proportion

^b^ Cut-offs were varied varied depending on location. Cut-off was based on the province-specific median proportion of bed net ownership [4]

^c^At least 60% of villagers had access to improved drinking water sources (tap/piped water, boreholes, protected dug wells, protected springs and rainwater collection). Cut-off was determined based on national coverage [4]

^d^ Cut-offs were province-specific; the average proportion of population age 15 years or more attained high-school degree based on Provincial Statistical reports

**Fig. 2:**
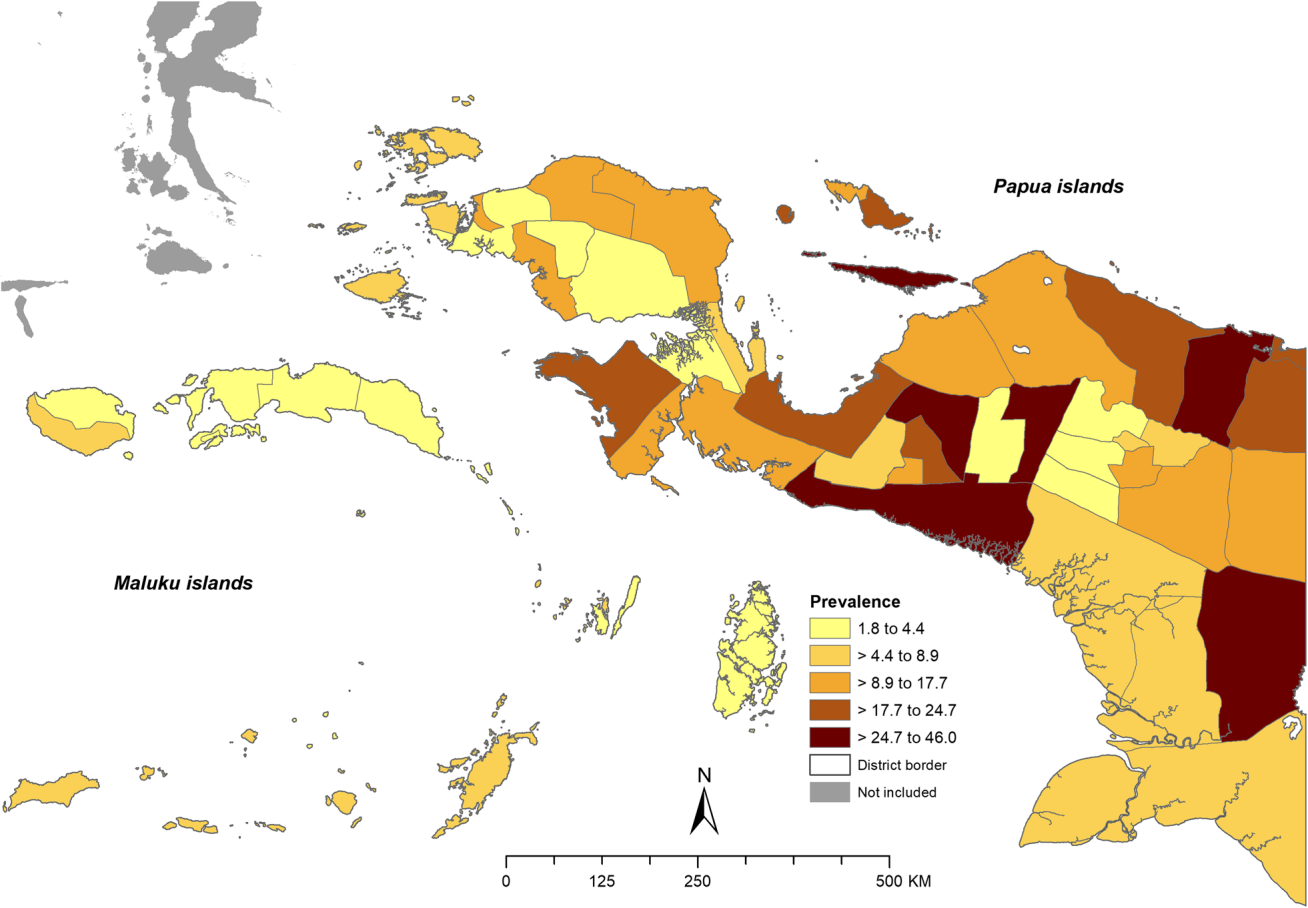
Self-reported malaria prevalence in adults ≥ 15 years of age at district-level in Maluku, West Papua and Papua province, Indonesia

Among all surveyed adults in Maluku, West Papua and Papua, 4.1%, 12.4% and 18.8%, respectively, reported a history of having laboratory confirmed malaria in the last 12 months. Of which, the majority of respondents in Maluku (54.4%) were not known the type of infection. While in both West Papua and Papua, larger proportion of respondents (56.9% and 41%, respectively) were diagnosed of having *P. vivax* infection. Figure 2 shows self-reported malaria prevalence at district-level. High self-reported malaria prevalence was observed in five districts in Papua province including Jayapura (38.2%), Puncak Jaya (36.1%), Mimika (33.1%), Boven Digoel (30.8%) and Intan Jaya (22.4%). In West Papua province, the highest self-reported malaria prevalence was found in Fakfak. In Maluku, the prevalence was ranged from 2.6 to 6.9%, with the highest identified in Maluku Barat Daya district.

### Risk factors of reporting malaria in the past 12 months

The results of the bivariate analysis for all three provinces are presented in Table 2. Bivariable logistic regression showed that gender was statistically associated with self-reported malaria (Maluku, *P *< 0.001), revealing that males are likely to self-reporting malaria (OR = 1.47, 95% CI 1.24–1.75). No statistically significant associations were observed between gender and self-report malaria in both West Papua (*P *= 0.493) and Papua (*P *= 0.591). Occupation, education and slept under a net last night before the survey were statistically associated with self-reporting malaria in all study sites. In the final multivariate multilevel analysis, odds of a malaria self-report were much higher for men (OR = 1.51, 95% CI 1.37–1.67) compared with women (Maluku) (Table 3). Farmers both in Maluku (OR = 1.31, 95% CI 1.16–1.48) and Papua (OR = 1.17, 95% CI 1.09–1.25) were likely to self-reported malaria relative to other occupational groups. Whilst in West Papua, the odds of reporting malaria were much higher (OR = 1.12, 95% CI 1.02–1.23) for adults who worked in tertiary sectors. In Maluku, adults who had attained primary (OR = 0.66, 95% CI 0.52–0.83) and secondary education (OR = 0.67, 95% CI 0.53–0.83) were less likely to self-report malaria relative to those who had no education. In contrast, in Papua, adults who had either primary (OR = 1.33, 95% CI 1.20–1.47) or secondary (OR = 1.27, 95% CI 1.15–1.40) degrees were more likely to self-report malaria compared to those who were not school. Odds of a malaria self-report were much lower for adults that slept using mosquito net a night before the survey (OR = 0.88, 95% CI 0.82–0.94) (Papua) relative to those participants who did not use ITN.

**Table 2:** Univariate analysis (odds ratio [OR] with 95% confidence interval [95% CI]) of the predictors of self-reporting malaria among adults aged ≥ 15 years in Maluku, West Papua and Papua

Factors	Maluku			West Papua			Papua		
	n/N	OR^a^ (95% CI^b^)	P-value	n/N	OR (95% CI)	P-value	n/N	OR (95% CI)	P-value
Gender			<*0.001*			0.493			0.591
Female	249/6378	1		479/4150	1		1931/10,225	1	
Male	314/5541	1.47 (1.24–1.75)		426/3853	0.95 (0.83–1.09)		2033/10,932	0.98 (0.91–1.04)	
Occupation			*0.040*			*0.027*			*<0.001*
Not working	226/5709	1		416/3705	1		1358/7067	1	
Primary industry workers (farmer, fisherman)	206/3383	1.57 (1.29–1.90)		177/1980	0.77 (0.63–0.95)		1520/9358	0.81 (0.73–0.90)	
Tertiary industry workers (services-related occupation)	103/2273	1.15 (0.90–1.46)		275/2033	1.23 (1.05–1.46)		903/3902	1.26 (1.13–1.41)	
Other (undefined)	28/554	1.29 (0.86–1.93)		37/285	1.17 (0.80–1.73)		183/830	1.18 (0.98–1.43)	
Education			*0.025*			*<0.001*			*<0.001*
No	38/532	1		50/579	1		555/4481	1	
Primary	217/4501	0.65 (0.45–0.95)		325/3224	1.18 (0.86–1.63)		1397/7438	1.63 (1.42–1.88)	
Secondary	308/6886	0.60 (0.42–0.87)		530/4200	1.53 (1.10–2.11)		2012/9238	1.96 (1.71–2.26)	
Slept using ITN last night			*<0.001*			*<0.001*			*0.223*
No	376/8840	1		509/3730	1		2577/13,858	1	
Yes	187/3079	1.45 (1.19–1.77)		396/4273	0.65 (0.54–0.78)		1387/7299	1.00 (0.91–1.10)	
Owned bed net			*<0.001*			*<0.001*			*0.003*
No	361/8563	1		491/3710	1		2534/13,949	1	
Yes	202/3356	1.45 (1.20–1.76)		414/4293	0.69 (0.58–0.83)		1430/7208	1.11 (1.00–1.23)	
House density			0.721			*0.128*			*<0.001*
Less than 8/m^2^	166/3435	1		176/1713	1		1410/8449	1	
More than 8/m^2^	397/8484	0.96 (0.79–1.18)		729/6290	1.14 (0.93–1.40)		2554/12,708	1.25 (1.12–1.39)	
Access to nearest PHCs			*0.246*			*<0.001*			*<0.001*
< 30 min	438/9377	1		788/6680	1		2671/12,549	1	
Not available	42/1035	0.86 (0.61–1.21)		74/670	0.93 (0.66–1.29)		327/1753	0.84 (0.70–1.01)	
> 30 min	83/1507	1.18 (0.89–1.59)		43/653	0.53 (0.36–0.76)		966/6855	0.60 (0.53–0.69)	
Household wealth index			*0.001*			*<0.001*			*<0.001*
Poorest	122/2129	1		251/2893	1		1955/12,902	1	
Poorer	120/2253	0.92 (0.70–1.22)		208/2118	1.14 (0.90–1.45)		582/2717	1.52 (1.32–1.75)	
Middle	108/2334	0.79 (0.59–1.06)		152/1169	1.57 (1.21–2.03)		457/1864	1.81 (1.56–2.11)	
Richer	105/2508	0.71 (0.53–0.96)		195/1143	2.16 (1.66–2.81)		513/1894	2.08 (1.73–2.49)	
Richest	108/2695	0.68 (0.50–0.92)		99/680	1.79 (1.33–2.41)		457/1780	1.93 (1.58–2.35)	
Proportion of villagers with improved access to safe drinking water^a^			*<0.001*			*<0.001*			*<0.001*
Low	304	1		153	1		1612	1	
High	259	0.67 (0.54–0.82)		752	1.39 (1.07–1.81)		2352	1.58 (1.39–1.78)	
Place of residence			*0.001*			*<0.001*			*<0.001*
Urban	188/4786	1		302/2053	1		1335/5435	1	
Rural	375/7133	1.36 (1.08–1.69)		603/5950	0.65 (0.53–0.79)		2629/15,722	0.61 (0.53–0.71)	
Proportion of villagers with had secondary education degree^b^			*0.010*			*<0.001*			*<0.001*
Low	254/4756	1		271/3170	1		1239/8608	1	
High	309/7163	0.79 (0.65–0.98)		634/4833	1.61 (1.30–2.00)		2725/12,549	1.64 (1.44–1.88)	
Proportion of villagers with bed net^c^			*<0.001*			*<0.001*			*<0.001*
Low	274/6667	1		529/3505	1		1930/11,011	1	
High	289/5252	1.36 (1.10–1.67)		376/4498	0.51 (0.42–0.62)		2034/10,146	1.17 (1.04–1.32)	
Elevation			0.882			*<0.001*			*<0.001*
Midland (200–1200 m)	290/6103	1		192/2151	1		1920/8546	1	
Lowland (< 200 m)	273/5816	0.98 (0.80–1.21)		510/4704	1.24 (0.99–1.54)		787/5200	0.61 (0.53–0.70)	
Upper highland (> 1200 m)	–	–		203/1148	2.19 (1.56–3.07)		1257/7411	0.70 (0.61–0.80)	

Self-reported malaria defined when respondent had malaria within the last 12-months and diagnosed by local health providers. Italic value indicates a statistically significant association at *p*-value less than 0.25

^a^OR, Odd ratio

^b^95% CI, 95% confidence interval

**Table 3:** Measures of association and variation between individual, household- and community-level factors and self-reported malaria in adults aged ≥ 15 years in three provinces, Indonesia

Variable	Maluku OR (95% CI)	West Papua OR (95% CI)	Papua OR (95% CI)
Fixed effect part			
Individual/household-level			
Gender			
Male	*1.51 (1.37–1.67)*	NA	NA
Female	Ref	NA	NA
Occupation			
Not working	Ref	Ref	Ref
Primary industry workers (farmer, fisherman)	*1.29 (1.15–1.46)*	1.01 (0.91–1.12)	*1.17 (1.09–1.25)*
Tertiary industry workers (services-related occupation)	*1.15 (1.00–1.32)*	*1.12 (1.02–1.23)*	1.05 (0.98–1.12)
Other (undefined)	*1.28 (1.01–1.61)*	1.04 (0.87–1.25)	1.09 (0.96–1.24)
Education			
No	Ref	Ref	Ref
Primary	*0.66 (0.52–0.83)*	1.11 (0.94–1.31)	*1.33 (1.20–1.47)*
Secondary	*0.67 (0.53–0.83)*	1.04 (0.87–1.24)	*1.27 (1.15–1.40)*
Slept using ITN last night			
No	Ref	Ref	Ref
Yes	1.11 (0.97–1.27)	0.96 (0.87–1.07)	*0.88 (0.82–0.94)*
Bed net ownership			
No	Ref	Ref	Ref
Yes	*1.16 (1.02–1.30)*	*1.21 (1.09–1.34)*	*1.12 (1.02–1.23)*
House density			
Less than 8/m^2^	NA	Ref	Ref
More than 8/m^2^	NA	1.05 (0.94–1.18)	1.00 (0.94–1.07)
Access to nearest PHCs			
Not available	0.99 (0.84–1.19)	*0.80 (0.68–0.95)*	*0.86 (0.78–0.95)*
Less than 30 min	Ref	Ref	Ref
More than 30 min	*1.30 (1.01–1.67)*	*0.75 (0.58–0.98)*	0.95 (0.86–1.05)
Household wealth index			
Poorest	Ref	Ref	Ref
Poorer	1.05 (0.90–1.20)	0.95 (0.82–1.11)	1.03 (0.94–1.14)
Middle	0.96 (0.80–1.14)	1.16 (0.98–1.38)	1.13 (0.99–1.30)
Richer	0.93 (0.77–1.13)	*1.38 (1.17–1.65)*	*1.33 (1.11–1.57)*
Richest	0.99 (0.81–1.22)	1.18 (0.97–1.42)	1.01 (0.84–1.22)
Village-level			
Proportion of villagers with improved access to safe drinking water^a^			
Low	Ref	Ref	Ref
High	*0.70 (0.60–0.83)*	0.94 (0.71–1.24)	1.03 (0.84–1.26)
Place of residence			
Rural	*1.31 (1.12–1.54)*	*1.56 (1.17–2.07)*	*0.47 (0.38–0.59)*
Urban	Ref	Ref	Ref
Proportion of villagers with had secondary education degree^b^			
Low	Ref	Ref	Ref
High	1.07 (0.89–1.27)	*1.28 (1.02–1.63)*	*1.45 (1.23–1.72)*
Proportion of villagers with bed net^c^			
Low	Ref	Ref	Ref
High	1.16 (0.98–1.37)	*0.59 (0.45–0.77)*	*1.26 (1.05–1.51)*
Elevation			
Lowland (< 200 m)	NA	1.12 (0.91–1.37)	*0.55 (0.47–0.64)*
Midland (200–1200 m)	NA	Ref	Ref
Highland (> 1200 m)		1.36 (0.96–1.94)	0.83 (0.67–1.02)
Random-effect part			
Village-level variance (s.e)	0.535 (0.057)	1.345 (0.100)	2.923 (0.159)
Household-level variance	0.863 (0.118)	0.670 (0.083)	1.203 (0.066)
PCV^d^ (%)	− 10.68	− 5.68	− 12.03

Italic value indicates a statistically significant association at *p*-value less than 0.05. NA variable had *p*-value more than 0.25 at bivariate analysis

*OR* odds ratio, *CI* confidence interval, *s.e* standard error, *Ref* reference, *PCV* percent changes in variance, *ITN* insecticide-treated net, *PHC* public health centre

^a^At least 60% of villagers had access to improved drinking water sources (tap/piped water, boreholes, protected dug wells, protected springs and rainwater collection). Cut-off was determined based on national coverage [4]

^b^Cut-offs were province-specific; the average proportion of population age 15 years or more attained high-school degree based on Provincial Statistical reports

^c^ Cut-offs were varied varied depending on location. Cut-off was based on the province-specific median proportion of bed net ownership [4]

^d^PCV, percent change in village-level variance between the null model (Model 0) and full model (Model 3)

In the bivariate analysis, household-level covariates include bed net ownership, house density, access to public health centers (PHCs) and wealth index were found to be associated with self-reporting malaria. Participants whose households owned bed net were found to have higher odds of reporting malaria (Maluku: OR = 1.45, 95% CI 1.20–1.76; Papua: OR = 1.11, 95% CI 1.00–1.23). Whereas in West Papua, participants whose households owned bed net were less likely to reporting malaria (OR = 0.69, 95% CI 0.58–0.83). Significant association between household density and self-reporting malaria was only appeared in West Papua (*P *= 0.13) and Papua (*P *< 0.001). Access to health facilities having significant impact on self-reporting malaria among West Papuan and Papuan participants; participants who perceived that the nearest PHC could be reached in more than 30 min were less likely to report malaria (OR = 0.53, 95% CI 0.36–0.76 and OR = 0.60, 95% CI 0.53–0.69, respectively). In the multivariate multilevel analysis, of all provinces, the odds of reporting malaria were 12 to 21% higher for adult participants who lived within the household that owned bed nets compared with those adults who resided in household without bed nets. In Maluku, adults who perceived that they should travel for more than 30 min for reaching the nearest healthcare services were more likely to self-report malaria (OR = 1.30, 95% CI 1.01–1.67) relative to those who perceived less than 30 min to travel to available healthcare facilities. Whilst in both West Papua and Papua, the odds of a malaria self-report were much lower (OR = 0.80, 95% CI 0.68–0.95 and OR = 0.86, 95% CI 0.78–0.95, respectively) among adults who perceived that there were no health facilities nearby relative to those who perceived less than 30 min to travel to available healthcare facilities. Participants in West Papua and Papua province were more likely to self-report malaria if living in a wealthier socioeconomic condition (OR = 1.38, 95% CI 1.17–1.65 and OR = 1.33, 95% CI 1.11–1.57, respectively) compared to those poorest households.

Bivariate analysis showed that village-level covariates include community education level, community bed net ownerships, community access to improved drinking water and type of the village were found to have significant association (*P *< 0.05) with reporting malaria in all provinces. However, elevation was only statistically associated with self-report malaria in only West Papua (*P *< 0.001) and Papua (*P *< 0.001). The final multivariate analysis indicated that odds of reporting malaria were associated with access to safe drinking water, place of residence, community education, bed net coverage and zone. In Maluku, adults who were living in a village with better water supply (OR = 0.70, 95% CI 0.60–0.83) were less likely to self-report malaria relative to those who were living in poor drinking water supply. No significant association between access to improved drinking water source and self-report malaria in both West Papua and Papua. Odds of a malaria self-report were much higher among adults who living in rural areas (Maluku, OR = 1.31, 95% CI 1.12–1.54; West Papua, OR = 1.56, 95% CI 1.17–2.07). In contrast, in Papua, adults residing in rural areas were less likely to self-report malaria (OR = 0.47, 95% CI 0.38–0.59) relative to those who lived in urban areas. In West Papua and Papua, adults were more likely to self-report malaria if living in a village with better education level (OR = 1.28, 95% CI 1.02–1.63 and OR = 1.45, 95% CI 1.23–1.72, respectively) relative to those who lived in a village with less people who had attained at least secondary education. Adults living in a community where most people used bed net were less likely to self-report malaria (OR = 0.59, 95% CI 0.45–0.77) compared with those who lived in a community where less people used bed net (West Papua). In contrast, in Papua, people living in the community where majority of villagers using bed net were likely to report malaria (OR = 1.26, 95% CI 1.05–1.51). The odds of self-reported malaria were lower for those who lived in lowland (OR = 0.55, 95% CI 0.47–0.64) (Papua) relative to those who lived in the midland (200–1200 m). No association was apparent between self-reporting malaria and zone in both Maluku and West Papua.

In all province, the village-level variance decreased as village-level factor included in the model (Additional file 1: Tables S1–S3). The percent change in variance (PCV) was varied among provinces ranged from 5.68% (West Papua) to 12.03% (Papua).

## Discussion

This study has revealed individual, household and village-level factors associated with self-reported malaria in Maluku, West Papua and Papua province, Indonesia. This study demonstrated notable differences in factors associated with self-reported malaria at every level among provinces, suggesting the needs of further site-specific and targeted intervention programmes. This is the first study that provide evidence concerning self-reported malaria prevalence in adults and its associated factors in multi-locations in Indonesia.

When examining associations between individual-level risk factors and self-reported malaria, we identified that self-reporting malaria was associated with gender, occupation and education although there were differences between locations. The odds of reporting malaria tended to be higher in adults who worked in the primary industries (Maluku and Papua) and tertiary industries (West Papua). The finding that people who work in the primary sectors such as farmers, fishermen and miners were more likely to report malaria might be relevant with the ecological setting that exists in Maluku and Papua. Coastal ecosystems, lowland rice fields and highland forests are common in these islands, which provide an ideal habitat for several malaria vectors, such as *Anopheles farauti*, *Anopheles bancroftii*, *Anopheles karwari*, *Anopheles koliensis* and *Anopheles punctulatus* [34].

Improving knowledge and awareness on malaria risk among rural communities should be the primary goal for these areas. Therefore, preventive interventions need a strong multi-sectoral collaboration between health and agriculture authorities. Differently, intervention strategies in West Papua should be directed to scaling-up malaria preventive campaigns toward tertiary workforces (e.g., civil servants, private workers, entrepreneurs) by involving local industries. It is also worth noting that Papuan adults who had better educational background tend to self-reporting malaria, but not in Maluku. This difference could be explained by the fact that people who have better education would normally be more knowledgeable and aware of malaria and thus could be more likely to either over-report of having malaria (Papua) or be aware of malaria prevention practices (Maluku). Other possible reasons could be explained by the fact that in such high transmission area with a considerable well-educated migrant population like in Papua, it was logical that adults with higher education status are more likely to report malaria. In both West Papua and Papua, the association of gender and self-reported malaria was negligible which suggest that in highly endemic areas both men and women could have even probability of acquiring malaria. Variation in evidence regarding associations between gender, occupation and education with malaria however have also been reported in several studies from Congo [12], India [35], Malawi [36] and Cambodia [37]. These findings suggest that different strategies for malaria prevention and control should be targeted towards different socio-demographical groups in each province, such as approaches to promote awareness on malaria in Maluku could be differ from that in Papua.

The use of ITN a night before the survey is associated with self-reporting malaria among Papuan adults; which those who used ITN were less likely to report malaria at the time of the survey. This finding is inconsistent with the previous study in Papua [19], which found that ITN has a positive correlation with acquiring malaria; those who slept under a net was likely to have higher odds of malaria. This may be due to discrepancy in sample included in the analysis since this study only included adults aged 15 years above while the previous study [19] included all age-groups in their analysis. The current study also revealed intriguing evidence on the association between self-reported malaria and bed net ownership. The findings showed that the odds for reporting malaria was 12–21% higher in that adults who owning bed net. When self-report malaria is used as the measure of malaria infection, possible explanations for this might be due to low compliance level among adults on utilizing and retreating the net to prevent mosquito bites and maintain the efficacy of bed net as well as people’s habits which is likely influenced by their awareness and belief regarding malaria [17, 38, 39]. Studies in Kenya have demonstrated that individuals’ attitude and views and social cultural norms influenced the use of nets among bed net owners [39]. A report from North Maluku could partly explain this finding that there was a common behaviour among adults in this region to regularly go outside at night or sleep outside for several reasons (e.g., watching television, social gatherings or fishing) without protection leading to high risk of malaria transmission [17]. In addition to human behaviour, local vector behaviour could be also other important factor affecting the effectiveness of bed net. For instance, *An. punctulatus* which is a common malaria vector in Maluku and Papua that usually bite humans outdoors. Additionally, there is other malaria vector such as *An. farauti* which has also been found to have strong exophagic preference and peak biting activity in early evening [34], where most people are still awake and less likely to use bed net. Indeed, this finding suggests that whilst Indonesian government has massively distributed bed nets towards these high transmission provinces, its utilization and factors associated with the effectiveness and compliance on using bed nets are far from clear and thus further studies should measure bed net utilization and retreatment among these populations to better inform health authorities on designing effective interventions. Targeted measures have to be applied towards these communities through sensitizing the community on re-treating their nets regularly to improve the effectiveness of bed nets and promoting the use of topical repellents.

The association between household wealth status and reporting malaria was observed in West Papua and Papua province. The study demonstrated that people lived in more affluent (richer) households more likely to reporting malaria compared to those individuals who lived in the poorest households. Whereas, there was no apparent association between self-reported malaria and household’s wealth in Maluku. When self-report malaria is used as the measure of malaria infection, these findings may be contradicting with general agreements given that poor communities is prone to contracting malaria [12, 40–43] although the relationship between malaria and poverty at the micro-level (e.g., household and population) has been reportedly varied across studies [44]. However, the results of this study are supported by the findings of other study in Nigeria where most malaria contracted by better-off socioeconomic status and urban populations [45]. Whereas others [46, 47] also found no clear association between household socioeconomic status and malaria. The fact that the odds of reporting malaria was higher among wealthy and well-educated populations in Papua and West Papua could perhaps partly explained by a great number of migrants come from outside Papua with better socioeconomic status that came to works (i.e., gold miners) in this region. It is known that there is the largest gold mining in Papua. It is worth noting that illness awareness and perception among wealthier and well-educated people would normally better than those poor people. These richer groups have better capability in recognizing symptoms and access in seeking treatment immediately and hence they are likely to report malaria. However, further epidemiological studies are needed to understand the relationships between socioeconomic status and actual malaria illness since using self-report malaria could be over (under) report infection [47].

The results of this study also indicated that distance in terms of time spent to travel from home to the nearest health facility was significantly associated with reporting malaria in all sites. In Maluku, individuals who spent longer time to travel (> 30 min) from their home to reach the nearest public health centres (PHCs) tend to have 30% greater odds of reporting malaria. The result was consistent with other observations in Tanzania [48] and India [35]. If those people who self-reported malaria were assumed of having true positive malaria, this finding explain that indeed delays in seeking health services to obtain appropriate and timely treatments is one of the factors that may exacerbate the risk of malaria. Extreme geographical conditions and poor infrastructures (e.g., road and transportation) may likely preventing people for having timely treatments especially for those who live in remote rural areas. However, different responses were observed in both West Papua and Papua, which the odds of reporting malaria was lower among those who perceived that there was no health facility available in their neighbourhood. Such findings could be explained by the fact that this self-reported might not be correlated with malaria infection and it might be that people who live in the area where there is no point-of-care available or even those who live further away are less likely to seek treatment and hence less likely to reporting malaria. Taken together, limited health services and supporting physical infrastructures could affect their behaviour in seeking treatments which could either lead to greater risk of infection or under-reporting malaria illness. These findings highlight the importance of both strengthening active community-based interventions and strong inter-sectoral collaboration (i.e., health, rural and public works department) to provide equal access to health services and enhanced active surveillance.

This study also highlights the influence of factors operating at the contextual level regard to self-reporting malaria in adults. At the village level, in all provinces, the study showed that self-reporting malaria was associated with the type of residential. Adults in rural Maluku and West Papua were likely to report malaria compared to those who resided in urban areas. In addition, those participants in whose village had better water infrastructure seem to less likely to reporting malaria. These findings are consistent with a typical condition where malaria transmission commonly found in such disadvantaged and deprived rural communities [19]. If self-report malaria is measured as malaria incidence, one reasonable explanation for this is that rural people in Maluku and West Papua who have no adequate access to improved drinking water sources may likely to frequently travel to collect water in the river or spring in the forest, which may increase the likelihood of exposure to mosquito biting such as *An. punctulatus* and *An. farauti*.

These findings highlight needs of interventions to positively improve access on drinking water sources in rural areas. In addition, other explanation for these may be due to the fact that rural households in Maluku and West Papua are tend to keep and raise livestock (e.g., cattle, pigs) around their house, which potentially attract zoophilic *Anophelines* species such as *An. koliensis* and *An. farauti* and hence facilitate higher transmission [49]. However, data for the presence or livestock ownership were not collected in the survey. Different response was observed in Papua; Papuan people who lived in rural areas appears to less likely to report malaria compared to those who lived in the cities. It is possible that this self-reported malaria may be influenced by factors including the availability of health facilities and diagnostic services in the village.

Community education level was associated with self-reporting malaria in both West Papua and Papua. Notably, in both West Papua and Papua, people who living in the community where the majority of residents are well-educated more likely to report malaria. That said, these findings suggest that self-reported malaria may be due to the fact that people were more aware of malaria, hence people were more likely to report malaria. Community bed net ownership was found to have different effects on self-reporting malaria in West Papua and Papua. Community bed net ownership decreased the odds of self-reporting malaria in West Papua but contrarily it increased the odds of self-reporting malaria in Papua. The findings in West Papua highlight that the community bed net ownership at the community level more greatly decreases of having malaria that at the household level; suggesting that the effect of community on malaria transmission is important. In contrast, in Papua, community bed net ownership may less effective relative to individual level bed net ownership. Possible explanation for this observed association includes herd immunity that may be exist within communities. The existence of individuals with asymptomatic and sub-microscopic infections within household or community may facilitate malaria transmission and reduce the mass effect of bed net [50]. A recent study in Timika city Papua have showed a considerable proportion of asymptomatic infections among older Papuans, females and those who did not own bed net [51]. This study also revealed associations between elevation and self-reported malaria in Papua in which the odds of reporting malaria was greatly lower among those who lived in lowland (< 200 m) relative to those who lived in the midland (200–1200 m). Contrastingly, the odds of self-reporting malaria among West Papuan people resided in lowland (< 200 m) and highland (> 1200 m) was greatly higher compared to those who lived in midland, although the association was not statistically significant. It is possible that this self-reported malaria in Papua may not correlated with malaria infection and it may be influenced by factors include awareness, socioeconomic condition and access to healthcare services. While if self-reporting malaria is used as measure of malaria infection, the evidence from West Papua could be explained due to the fact the existence of malaria vectors and their habitats in all elevation level. Three major malaria vectors in Papua include *An. punctulatus*, *An. koliensis* and *An. farauti* have been found in lowland and highlands of Papua (> 1200 m) [34, 52].

## Limitations

The present study has several limitations thus the findings should be carefully interpreted. Firstly, it is worth noting that the prevalence of self-report malaria reported in this survey may not reflect the actual estimates of malaria prevalence in the areas studied as the data used in this study are declarative based on individual’s experience without validation through laboratory examination, but it already provides reliable important data. However, this study did not able to compare self-reported malaria estimates with the actual malaria prevalence, especially in these three provinces as there are no recent malaria prevalence data available and no comparable large-scale population-based surveys representative of district- or province-level have been conducted so far. Hence, there is a need to verify the recent prevalence of malaria with a more comprehensive population-based standalone malariometric survey to provide guidance for policymaking and elimination malaria efforts in these three provinces. To ascertain that self-report approach used in this study could reflect general malaria condition in the areas studied, further separate assessment to compare self-reported malaria prevalence against notified laboratory-confirmed malaria incidence was carried out (Additional file 2: Figure S1) The analysis revealed a striking evidence that there is a consistent trend and strong correlation (Spearman’s rho = 0.9) between self-reported malaria prevalence obtained from the RISKESDAS 2013 survey and malaria incidence at province-level across Indonesia in the previous years prior to the survey. This indicates that self-reported (questionnaire) approach has been able to adequately capture the variation in magnitude of malaria transmission at province-level and hence it is still appropriate and reasonable for use in studies on investigating risk factors associated with malaria. On top of that, the diagnostic capacity to perform unified microscopic procedure in every public health centres (PHCs) and active case finding have been improved in the past decade especially in these hyperendemic areas studied [2], so it is possible that this may eliminate potential bias associated with self-reporting malaria (e.g., that the respondents had truly been diagnosed of having malaria). It is important to note that the data used in this study were a subset of National Basic Health Research which conducted by the Ministry of Health of Indonesia. It is a large-scale population-based survey aiming to provide policymakers with recent evidence based and reliable information on public health indicators representative of the national, provincial, district-level include non-communicable and communicable diseases, pharmacy and traditional medicine, access and health services, oral health, mental health, health financing, reproductive health, children’s health, injury, disability, housing and economic, health behaviours and practices. Given the nature of this survey, there were logistical and financial constraints which limited the data collection (e.g., the use of RDT to confirm malaria). Thus, self-report of malaria is the easy and feasible way to capture general malaria status across Indonesia archipelago. However, recall bias and social desirability bias may be still existed due to self-report and thus affecting responses and leading to under (over) report of malaria or ITN/bed net utilization in population [53, 54]. This study suggests that malaria diagnostic testing should be performed in the future RISKESDAS surveys to provide better information on malaria prevalence and minimise the biases. Despite its limitations, self-reported malaria however has also been used in several African studies [45, 55–57] as well as Indonesian studies [19, 49] and it has helped provide important insights on the epidemiology of malaria in the region. Secondly, as this study used data from the cross-sectional survey, this study did not able to explain causal relationships underlying the malaria infection in the areas studied; however the present study was not aimed to make any causal claims. In addition, the results of this study might be also influenced by the effects of recall biases as we utilized self-reported data. Moreover, the multilevel models also indicated that according to the PCV there might be some unmeasured contextual factors at the village-level that could better explain the variation in the village-level in association with the probability of malaria. Despite these shortcomings, this information would have been useful in further understanding the aetiology of malaria in area studied.

## Conclusions

This study revealed notable differences in individual and contextual factor of self-reported malaria between provinces in eastern Indonesia. This study demonstrated the importance of directing more prevention and control efforts towards the communities in Maluku, West Papua and Papua. In addition, the findings highlight the need to strengthen the implementation of multi-sectoral collaboration between health authorities and related stakeholders (e.g., bureau of education, bureau of public works and infrastructure) to improve designs in planning and interventions strategies for reducing malaria burden in Maluku, West Papua and Papua province. The prevalence of self-report malaria reported in this survey may not reflect the actual malaria infection prevalence. However, this study provided important insights of the epidemiology of malaria in diverse provinces in Indonesia as well as provide recommendation for future research.

## Additional files

**Additional file 1: Table S1.** Parameter estimates from multilevel models of self-reported malaria, Maluku. **Table S2.** Parameter estimates from multilevel models of self-reported malaria, West Papua. **Table S3.** Parameter estimates from multilevel models of self-reported malaria, Papua.

**Additional file 2: Figure S1.** Comparison between self-reported malaria prevalence (RISKESDAS 2013) and notified laboratory confirmed malaria morbidity (Annual Parasite Incidence per 1000 persons) in 2012 across 33 provinces in Indonesia. The chart demonstrated a strong signal of correlation between self-reported prevalence and malaria incidence one year before the survey (Spearman’s rho = 0.906, *P* value = 0.001) as well as the mean incidence of period 2009–2012 (Spearman’s rho = 0.913, *P* value = 0.001) in each province. This indicated that self-reported malaria prevalence resulted from the survey tends to reflect the incidence of malaria in every province.

## Abbreviations

RISKESDAS: Riset Kesehatan Dasar
OR: odd ratio
CI: confidence interval
API: annual parasite incidence
ITNs: insecticide-treated nets
LLINs: long-lasting insecticidal nets
PSU: primary sampling unit
CB: census block
KAP: knowledge-attitude-practice
RDT: rapid diagnostic test
DEM: digital elevation model
PCA: principle component analysis
PCV: proportional change in variance
PHC: public health centre
