# Supplementary material for: Individual and contextual factors predicting self-reported malaria among adults in eastern Indonesia: findings from Indonesian community-based survey
Source: Malar J. 2019 Apr 4;18:118. doi: 10.1186/s12936-019-2758-2 (PMC6449936; doi:10.1186/s12936-019-2758-2)
Supplement: Supplementary file 1 — Additional file 1: Table S1. Parameter estimates from multilevel models of self-reported malaria, Maluku. Table S2. Parameter estimates from multilevel models of self-reported malaria, West Papua. Table S3. Parameter estimates from multilevel models of self-reported malaria, Papua. [file 12936_2019_2758_MOESM1_ESM.docx]

**Additional file 1**

**Table S1.** Parameter estimates from multilevel models of self-reported malaria, Maluku (n=11,919)

| **Characteristic** | **Model 0** | **Model 1** | **Model 2** | **Model 3** |
| --- | --- | --- | --- | --- |
|  | **OR (95% CI)** | **OR (95% CI)** | **OR (95% CI)** | **OR (95% CI)** |
|  |  |  |  |  |
| **Individual-level** |  |  |  |  |
| *Gender* |  |  |  |  |
| Male |  | **1.51 (1.36-1.67)** | **1.51 (1.37-1.67)** | **1.51 (1.37-1.67)** |
| Female |  | Ref | Ref | Ref |
| *Occupation* |  |  |  |  |
| Not working |  | Ref | Ref | Ref |
| Primary industry workers (farmer, fisherman) |  | **1.37 (1.21-1.55)** | **1.33 (1.17-1.50)** | **1.29 (1.15-1.46)** |
| Tertiary industry workers (services-related occupation) |  | 1.10 (0.97-1.26) | 1.13 (0.99-1.30) | **1.15 (1.00-1.32)** |
| Other (undefined) |  | **1.26 (1.00-1.59)** | 1.26 (0.99-1.59) | **1.28 (1.01-1.61)** |
| *Education* |  |  |  |  |
| No |  | Ref | Ref | Ref |
| Primary |  | **0.64 (0.52-0.80)** | **0.65 (0.52-0.82)** | **0.66 (0.52-0.83)** |
| Secondary |  | **0.63 (0.51-0.79)** | **0.66 (0.53-0.83)** | **0.67 (0.53-0.83)** |
| *Used ITN last night* |  |  |  |  |
| No |  | Ref | Ref | Ref |
| Yes |  | **1.23 (1.11-1.37)** | 1.19 (0.98-1.27) | 1.11 (0.97-1.27) |
|  |  |  |  |  |
| **Household-level** |  |  |  |  |
| *Bednet ownership* |  |  |  |  |
| No |  |  | Ref | Ref |
| Yes |  |  | **1.19 (1.06-1.34)** | **1.16 (1.02-1.30)** |
| Household-density |  |  |  |  |
| Low |  |  | NA | NA |
| High |  |  | NA | NA |
| *Access to nearest PHCs* |  |  |  |  |
| Not available |  |  | 0.99 (0.84-1.19) | 0.98 (0.82-1.18) |
| Less than 30 minutes |  |  | Ref | Ref |
| More than 30 minutes |  |  | **1.35 (1.10-1.65)** | **1.30 (1.01-1.67)** |
| *Household wealth index* |  |  |  |  |
| Poorest |  |  | Ref | Ref |
| Poorer |  |  | 1.01 (0.87-1.18) | 1.05 (0.90-1.20) |
| Middle |  |  | 0.90 (0.76-1.07) | 0.96 (0.80-1.14) |
| Richer |  |  | 0.84 (0.70-1.00) | 0.93 (0.77-1.13) |
| Richest |  |  | 0.86 (0.72-1.04) | 0.99 (0.81-1.22) |
|  |  |  |  |  |
| **Village-level** |  |  |  |  |
| *Proportion of villagers attained 2^nd^ education level*^a^ |  |  |  |  |
| Low |  |  |  | Ref |
| High |  |  |  | 1.07 (0.89-1.27) |
| *Proportion of villagers used bed net*^b^ |  |  |  |  |
| Low |  |  |  | Ref |
| High |  |  |  | 1.16 (0.98-1.37) |
| *Proportion of villagers had access on improved water sources*^c^ |  |  |  |  |
| Low |  |  |  | Ref |
| High |  |  |  | **0.70 (0.60-0.83)** |
| *Place of residence* |  |  |  |  |
| Rural |  |  |  | **1.31 (1.12-1.54)** |
| Urban |  |  |  | Ref |
| *Zone* |  |  |  |  |
| Lowland (<200 m) |  |  |  | **1.21 (1.02-1.44)** |
| Midland (200-1200) |  |  |  | Ref |
| Upper Highland (>1200 m)* |  |  |  | - |
|  |  |  |  |  |
| Intercept (Coef) | -3.582 | -3.702 | -3.60 | -3.85 |
|  |  |  |  |  |
| *Random effect* |  |  |  |  |
| Village-level variance (s.e) | 0.599 (0.005) | 0.571 (0.061) | 0.579 (0.062) | 0.535 (0.056) |
| Household-level variance (s.e) | 0.828 (0.010) | 0.862 (0.117) | 0.860 (0.117) | 0.863 (0.118) |
| PCV^†^ (%) |  | -4.67 | -3.17 | -10.68 |

*Abbreviations*: OR, odds ratio; CI, confidence interval; s.e, standard error; Ref, reference; PCV, percent changes in variance; ITN, insecticide-treated net; PHC, public health center; NA, not applicable. ^a^at least 60% of villagers had access to improved drinking water sources (tap/piped water, boreholes, protected dug wells, protected springs and rainwater collection) [4]; ^b^at least 60% of population had attained a secondary degree [27]; ^c^Cut-off of 25% was used based on province-level coverage [4]. ^†^ PCV, percent change in village-level variance between the null model (Model 0) and full model (Model 3); **Bold value** indicates a statistically significant association at *p*-value less than 0.05. NA variable had p-value more than 0.25 at bivariate analysis. *no observation recorded in Upper Highland.

**Table S2.** Parameter estimates from multilevel models of self-reported malaria, West Papua (n=8003)

| **Characteristic** | **Model 0** | **Model 1** | **Model 2** | **Model 3** |
| --- | --- | --- | --- | --- |
|  | **OR (95% CI)** | **OR (95% CI)** | **OR (95% CI)** | **OR (95% CI)** |
|  |  |  |  |  |
| **Individual-level** |  |  |  |  |
| *Gender* |  |  |  |  |
| Male |  | NA | NA | NA |
| Female |  | NA | NA | NA |
| *Occupation* |  |  |  |  |
| Not working |  | Ref | Ref | Ref |
| Primary industry workers (farmer, fisherman) |  | 0.97 (0.87-1.08) | 0.99 (0.89-1.11) | 1.01 (0.91-1.12) |
| Tertiary industry workers (services-related occupation) |  | **1.14 (1.04-1.25)** | **1.11 (1.01-1.23)** | **1.12 (1.02-1.23)** |
| Other (undefined) |  | 1.01 (0.84-1.23) | 1.03 (0.85-1.24) | 1.04 (0.87-1.25) |
| *Education* |  |  |  |  |
| No |  | Ref | Ref | Ref |
| Primary |  | 1.12 (0.95-1.31) | 1.10 (0.94-1.30) | 1.11 (0.94-1.31) |
| Secondary |  | 1.09 (0.92-1.30) | 1.04 (0.87-1.24) | 1.04 (0.87-1.24) |
| *Used ITN last night* |  |  |  |  |
| No |  | Ref | Ref | Ref |
| Yes |  | 0.96 (0.87-1.06) | 0.94 (0.84-1.03) | 0.96 (0.87-1.07) |
|  |  |  |  |  |
| **Household-level** |  |  |  |  |
| *Bednet ownership* |  |  |  |  |
| No |  |  | Ref | Ref |
| Yes |  |  | **1.19 (1.07-1.32)** | **1.21 (1.09-1.34)** |
| Household-density |  |  |  |  |
| Low |  |  | Ref | Ref |
| High |  |  | 1.05 (0.94-1.17) | 1.05 (0.94-1.18) |
|  |  |  |  |  |
| *Access to nearest PHCs* |  |  |  |  |
| Not available |  |  | **0.78 (0.66-0.93)** | **0.80 (0.68-0.95)** |
| Less than 30 minutes |  |  | Ref | Ref |
| More than 30 minutes |  |  | **0.60 (0.48-0.74)** | **0.75 (0.58-0.98)** |
| *Household wealth index* |  |  |  |  |
| Poorest |  |  | Ref | Ref |
| Poorer |  |  | 0.99 (0.86-1.14) | 0.95 (0.82-1.11) |
| Middle |  |  | **1.28 (1.08-1.51)** | 1.16 (0.98-1.38) |
| Richer |  |  | **1.53 (1.29-1.81)** | **1.38 (1.17-1.65)** |
| Richest |  |  | **1.25 (1.03-1.50)** | 1.18 (0.97-1.42) |
|  |  |  |  |  |
| **Village-level** |  |  |  |  |
| *Proportion of villagers attained 2^nd^ education level* |  |  |  |  |
| Low |  |  |  | Ref |
| High |  |  |  | **1.28 (1.02-1.63)** |
| *Proportion of villagers used bed net* |  |  |  |  |
| Low |  |  |  | Ref |
| High |  |  |  | **0.59 (0.45-0.77)** |
| *Proportion of villagers had access on improved water sources* |  |  |  |  |
| Low |  |  |  | Ref |
| High |  |  |  | 0.94 (0.71-1.24) |
| *Place of residence* |  |  |  |  |
| Rural |  |  |  | **1.56 (1.17-2.07)** |
| Urban |  |  |  | Ref |
| *Zone* |  |  |  |  |
| Lowland (<200 m) |  |  |  | 1.12 (0.91-1.37) |
| Midland (200-1200) |  |  |  | Ref |
| Upper Highland (>1200 m) |  |  |  | 1.36 (0.96-1.94) |
|  |  |  |  |  |
| Intercept (Coef.) | -2.83 | -2.93 | -3.04 | -3.26 |
|  |  |  |  |  |
| *Random effect* |  |  |  |  |
| Village-level variance | 1.425 (0.229) | 1.402 (0.103) | 1.366 (0.100) | 1.345 (0.100) |
| Household-level variance | 0.713 (0.183) | 0.711 (0.080) | 0.688 (0.081) | 0.670 (0.083) |
| PCV^†^ |  | -1.61 | -4.35 | -5.68 |

*Abbreviations*: OR, odds ratio; CI, confidence interval; s.e, standard error; Ref, reference; PCV, percent changes in variance ITN, insecticide-treated net; PHC, public health center; NA, not applicable. ^a^ at least 60% of villagers had access to improved drinking water sources (tap/piped water, boreholes, protected dug wells, protected springs and rainwater collection) [4]; ^b^ at least 60% of population had attained a secondary degree [28]; ^c^ Cut-off of 44% was used based on province-level coverage [4]. ^†^ PCV, percent change in village-level variance between the null model (Model 0) and full model (Model 3); **Bold value** indicates a statistically significant association at *p*-value less than 0.05. NA variable had p-value more than 0.25 at bivariate analysis.

**Table S3.** Parameter estimates from multilevel models of self-reported malaria, Papua (n=21,157)

| **Characteristic** | **Model 0** | **Model 1** | **Model 2** | **Model 3** |
| --- | --- | --- | --- | --- |
|  | **OR (95% CI)** | **OR (95% CI)** | **OR (95% CI)** | **OR (95% CI)** |
|  |  |  |  |  |
| **Individual-level** |  |  |  |  |
| *Gender* |  |  |  |  |
| Male |  | NA | NA | NA |
| Female |  | NA | NA | NA |
| *Occupation* |  |  |  |  |
| Not working |  | Ref | Ref | Ref |
| Primary industry workers (farmer, fisherman) |  | **1.12 (1.04-1.20)** | **1.14 (1.07-1.22)** | **1.17 (1.09-1.25)** |
| Tertiary industry workers (services-related occupation) |  | **1.07 (1.00-1.15)** | 1.05 (0.98-1.12) | 1.05 (0.98-1.12) |
| Other (undefined) |  | 1.10 (0.97-1.25) | 1.09 (0.96-1.24) | 1.09 (0.96-1.24) |
| *Education* |  |  |  |  |
| No |  | Ref | Ref | Ref |
| Primary |  | **1.36 (1.23-1.50)** | **1.35 (1.23-1.49)** | **1.33 (1.20-1.47)** |
| Secondary |  | **1.34 (1.21-1.48)** | **1.31 (1.18-1.44)** | **1.27 (1.15-1.41)** |
| *Used ITN last night* |  |  |  |  |
| No |  | Ref | Ref | Ref |
| Yes |  | **0.90 (0.84-0.96)** | **0.88 (0.83-0.95)** | **0.88 (0.82-0.94)** |
|  |  |  |  |  |
| **Household-level** |  |  |  |  |
| *Bednet ownership* |  |  |  |  |
| No |  |  | Ref | Ref |
| Yes |  |  | **1.13 (1.04-1.24)** | **1.12 (1.02-1.23)** |
| Household-density |  |  |  |  |
| Low |  |  | Ref | Ref |
| High |  |  | 1.03 (0.96-1.10) | 1.01 (0.94-1.08) |
| *Access to nearest PHCs* |  |  |  |  |
| Not available |  |  | **0.84 (0.76-0.94)** | **0.86 (0.78-0.95)** |
| Less than 30 minutes |  |  | Ref | Ref |
| More than 30 minutes |  |  | **0.90 (0.82-0.99)** | 0.95 (0.86-1.05) |
| *Household wealth index* |  |  |  |  |
| Poorest |  |  | Ref | Ref |
| Poorer |  |  | **1.11 (1.01-1.23)** | 1.03 (0.94-1.14) |
| Middle |  |  | **1.27 (1.12-1.45)** | 1.13 (0.99-1.30) |
| Richer |  |  | **1.50 (1.27-1.77)** | **1.33 (1.11-1.57)** |
| Richest |  |  | 1.17 (0.97-1.41) | 1.01 (0.84-1.22) |
|  |  |  |  |  |
| **Village-level** |  |  |  |  |
| *Proportion of villagers attained 2^nd^ education level* |  |  |  |  |
| Low |  |  |  | Ref |
| High |  |  |  | **1.45 (1.23-1.72)** |
| *Proportion of villagers used bed net* |  |  |  |  |
| Low |  |  |  | Ref |
| High |  |  |  | **1.26 (1.05-1.51)** |
| *Proportion of villagers had access on improved water sources* |  |  |  |  |
| Low |  |  |  | Ref |
| High |  |  |  | **1.03 (0.84-1.26)** |
| *Place of residence* |  |  |  |  |
| Rural |  |  |  | **0.47 (0.38-0.59)** |
| Urban |  |  |  | Ref |
| *Zone* |  |  |  |  |
| Lowland (<200 m) |  |  |  | **0.55 (0.47-0.64)** |
| Midland (200-1200) |  |  |  | Ref |
| Upper Highland (>1200 m) |  |  |  | 0.83 (0.67-1.02) |
|  |  |  |  |  |
| Intercept (Coef.) | -2.51 | -2.78 | -2.97 | -2.32 |
|  |  |  |  |  |
| *Random effect* |  |  |  |  |
| Village-level variance | 3.323 (0.163) | 3.262 (0.166) | 3.104 (0.166) | 2.923(0.159) |
| Household-level variance | 1.205 (0.066) | 1.197 (0.066) | 1.206 (0.067) | 1.203 (0.066) |
| PCV^†^(%) |  | -1.83 | -6.59 | -12.03 |

*Abbreviations*: OR, odds ratio; CI, confidence interval; s.e, standard error; Ref, reference; PCV, percent changes in variance ITN, insecticide-treated net; PHC, public health center; NA, not applicable. ^a^at least 60% of villagers had access to improved drinking water sources (tap/piped water, boreholes, protected dug wells, protected springs and rainwater collection) [4]; ^b^at least 60% of population had attained a secondary degree [29]; ^c^Cut-off of 25% was used based on province-level coverage [4]. ^†^ PCV, percent change in village-level variance between the null model (Model 0) and full model (Model 3); **Bold value** indicates a statistically significant association at *p*-value less than 0.05. NA variable had p-value more than 0.25 at bivariate analysis.
