# Supplementary figures and images for: Individual and contextual factors predicting self-reported malaria among adults in eastern Indonesia: findings from Indonesian community-based survey
Source: Malar J. 2019 Apr 4;18:118. doi: 10.1186/s12936-019-2758-2 (PMC6449936; doi:10.1186/s12936-019-2758-2)

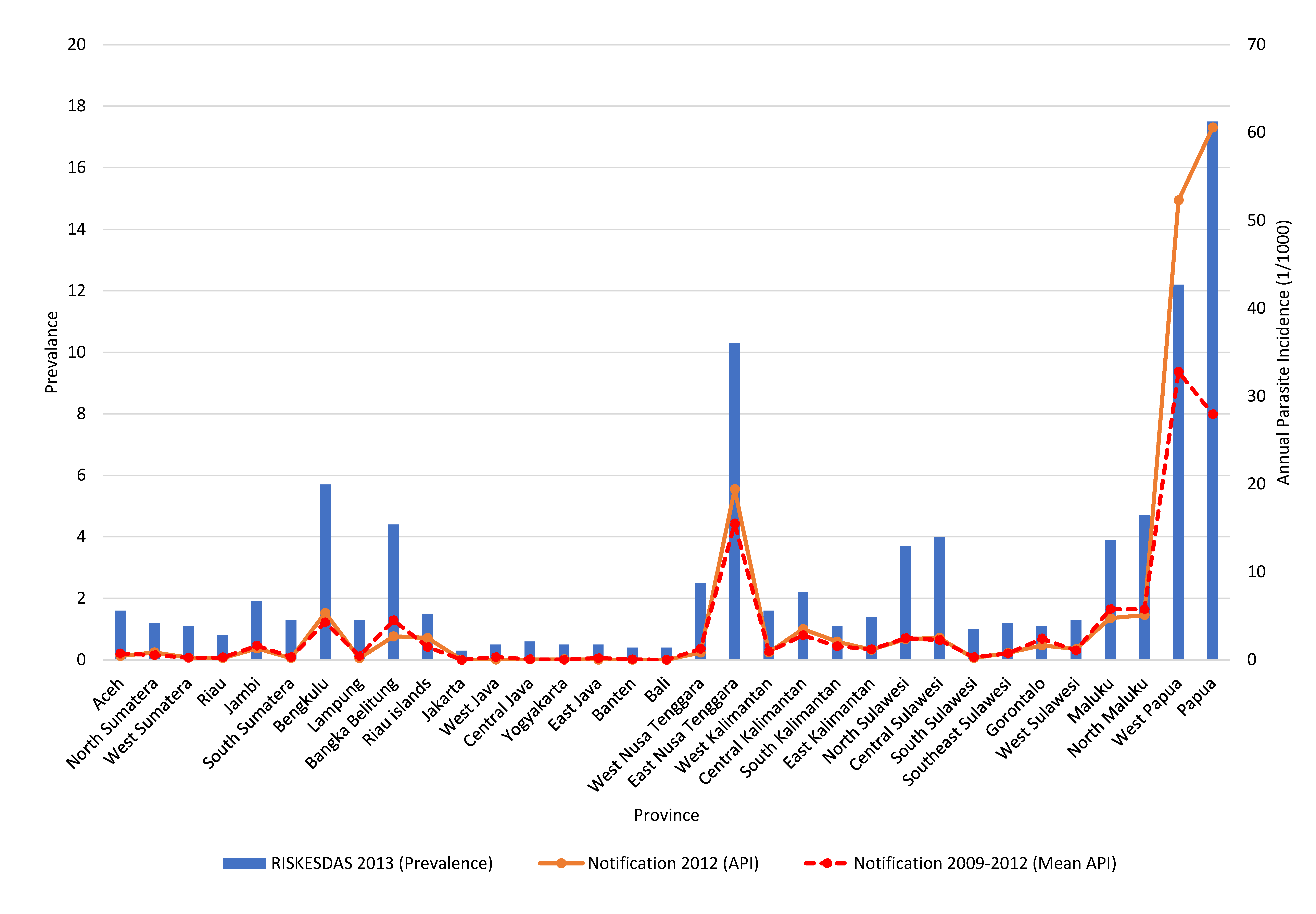

Supplement: Supplementary file 2 — Additional file 2: Figure S1. Comparison between self-reported malaria prevalence (RISKESDAS 2013) and notified laboratory confirmed malaria morbidity (Annual Parasite Incidence per 1000 persons) in 2012 across 33 provinces in Indonesia. The chart demonstrated a strong signal of correlation between self-reported prevalence and malaria incidence one year before the survey (Spearman’s rho = 0.906, P value = 0.001) as well as the mean incidence of period 2009–2012 (Spearman’s rho = 0.913, P value = 0.001) in each province. This indicated that self-reported malaria prevalence resulted from the survey tends to reflect the incidence of malaria in every province. [file 12936_2019_2758_MOESM2_ESM.tif]
